# MOF-Transformed In_2_O_3-*x*_@C Nanocorn Electrocatalyst for Efficient CO_2_ Reduction to HCOOH

**DOI:** 10.1007/s40820-022-00913-6

**Published:** 2022-08-17

**Authors:** Chen Qiu, Kun Qian, Jun Yu, Mingzi Sun, Shoufu Cao, Jinqiang Gao, Rongxing Yu, Lingzhe Fang, Youwei Yao, Xiaoqing Lu, Tao Li, Bolong Huang, Shihe Yang

**Affiliations:** 1 Guangdong Key Lab of Nano-Micro Material Research, School of Chemical Biology and Biotechnology, Peking University Shenzhen Graduate School, Shenzhen, 518055 People’s Republic of China; 2 Department of Chemistry and Biochemistry, Northern Illinois University, DeKalb, IL 60115 USA; 3 Department of Applied Biology and Chemical Technology, The Hong Kong Polytechnic University, Hung Hom, Kowloon, Hong Kong SAR People’s Republic of China; 4 School of Materials Science and Engineering, China University of Petroleum, Qingdao, Shandong 266580 People’s Republic of China; 5 Shenzhen International Graduate School, Tsinghua University, Shenzhen, People’s Republic of China; 6 X-Ray Science Division and Joint Center for Energy Storage Research, Argonne National Laboratory, Lemont, IL 60439 USA; 7 Institute of Biomedical Engineering, Shenzhen Bay Laboratory, Shenzhen, 518107 People’s Republic of China

**Keywords:** CO_2_ reduction, Indium oxide, Formate, Corn design, Active sites

## Abstract

For electrochemical CO_2_ reduction to HCOOH, an ongoing challenge is to design energy efficient electrocatalysts that can deliver a high HCOOH current density (*J*_HCOOH_) at a low overpotential. Indium oxide is good HCOOH production catalyst but with low conductivity. In this work, we report a unique corn design of In_2_O_3-*x*_@C nanocatalyst, wherein In_2_O_3-*x*_ nanocube as the fine grains dispersed uniformly on the carbon nanorod cob, resulting in the enhanced conductivity. Excellent performance is achieved with 84% Faradaic efficiency (FE) and 11 mA cm^−2^ *J*_HCOOH_ at a low potential of − 0.4 V versus RHE. At the current density of 100 mA cm^−2^, the applied potential remained stable for more than 120 h with the FE above 90%. Density functional theory calculations reveal that the abundant oxygen vacancy in In_2_O_3-*x*_ has exposed more In^3+^ sites with activated electroactivity, which facilitates the formation of HCOO* intermediate. Operando X-ray absorption spectroscopy also confirms In^3+^ as the active site and the key intermediate of HCOO* during the process of CO_2_ reduction to HCOOH.

**Supplementary Information:**

The online version contains supplementary material available at 10.1007/s40820-022-00913-6.

## Highlights

- The nanocorn design enables In_2_O_3-*x*_@C a high Faradaic efficiency of 98% and a high formate current density of 320 mA cm^−2^ at a low potential of -1.2 V versus hydrogen electrode without *iR* correction.
- The rich O vacancy activates In^3+^ sites on the nanocube shell of In_2_O_3-*x*_@C and the carbon cob enhances conductivity.
- Operando X-ray absorption spectroscopy unveils the active site of In^3+^ for formate production although reduction of In^3+^ to In is conceivable at the applied negative potentials during CO_2_ reduction reaction.

**Supplementary Information:**

The online version contains supplementary material available at 10.1007/s40820-022-00913-6.

## Graphical abstract

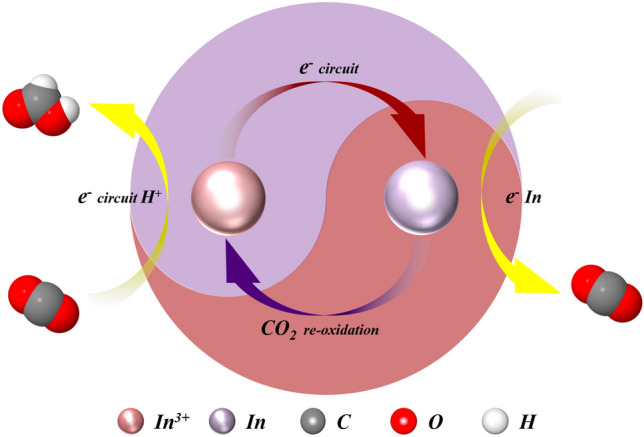

## Introduction

The alarmingly rising level of CO_2_ in the atmosphere has aggravated the greenhouse effect, raising serious concerns about the ecological, social, and sustainability problems across the globe. Electrochemical reduction of CO_2_ is a promising way to fulfill the carbon neutral goal and at the same time to generate valuable chemical feedstocks [1–8]. Among the various chemical products from CO_2_ reduction reaction (CO_2_RR), formic acid (HCOOH) is a key chemical of great industrial significance as well as an important hydrogen carrier for energy storage and conversion [9, 10]. However, the high thermal stability of CO_2_ and the multiple competing reaction pathways in CO_2_RR often result in a high overpotential and a low Faradaic efficiency (FE) for the production of formic acid through electrolysis [11]. Thus, it is imperative to design a suitable electrocatalyst to bring the activity and selectivity to an acceptable level for the production of formic acid.

The currently well-known catalysts for the formate generating CO_2_RR include indium (In)-, bismuth- and tin-based materials [12–24]. Although the highest FE of formic acid for In-based electrocatalysts has reached 95% [13, 14, 16], the overpotential is still high and the operating current density is generally low, which is partially induced by the conductivity limitation of the electrocatalysts. To our knowledge, the lowest potential that has been achieved at the current density of formate generation (~ 1 mA cm^−2^) was − 0.5 V versus reversible hydrogen electrode (RHE) with an FE below 80% [15]. Long-term stability performance of the electrocatalysts is essential, especially at large current densities (≥ 100 mA cm^−2^), but has rarely been reported [13, 14], and the longest tested time was less than 25 h at 140 mA cm^−2^ in 1 M KOH [14]. Oxygen vacancy in catalysts has shown beneficial roles in promoting the activation of CO_2_ for one thing and in accelerating electron transfer for another. However, how to maximize the advantages of vacancies still requires more delicate material designs [15, 25–29]. Due to the good conductivity, carbon paper and carbon cloth are commonly used to support powder electrocatalysts. One can envision that if such electrocatalysts could be supported and dispersed on nanoscale carbon uniformly, good conductivity and high catalyst loading will be simultaneously achieved, which also allows the optimal exploitation of the intrinsic activity of the catalysts.

On the fundamental side, the active sites of In-based electrocatalysts for converting CO_2_ to formic acid are still unclear; both In^3+^ and In were reported to be the active sites by different research groups [11, 13, 16]. The reduction of In^3+^ to In is conceivable under the applied negative potentials during CO_2_RR [13, 14], and a pertinent question remains on the correlation between the interplay among different oxidation states of In and the CO_2_RR performances. To elucidate the active sites and their action mechanism, performing operando experiments has become a necessity. While *in situ* Raman and Fourier transform infrared spectroscopy measurements have been reported on the In-based catalysts during the CO_2_RR, limited information has been obtained about the real active sites [13, 16, 30].

Herein, we propose a novel fine-grained corn design strategy of electrocatalysts and demonstrate its synthesis and catalytic performance using In_2_O_3-*x*_@C as a prototypical example. The corn-like nanostructure was obtained from a simple metal–organic framework (MOF)-based shape-preserving transformation process. The oxygen vacancies in In_2_O_3-*x*_ nanocubes densely and uniformly shelled on the in situ formed carbon cob support bring in the optimized electronic structures of the In active sites and the low charge transfer resistance, resulting in excellent activity for electrochemical reduction of CO_2_ to HCOOH. We obtained a high FE of 98% and a high HCOOH current density of 320 mA cm^−2^ at a low potential of −1.2 V versus RHE without *iR* correction. Moreover, operando X-ray absorption spectroscopy under a series of reaction potentials points to In^3+^ as the active sites for CO_2_ electrochemical reduction to HCOOH, but the catalyst was reduced to In briefly at negative potentials at the initial activation stage and then returned to In^3+^ to run the catalytic cycles.

## Experimental and Calculation

### Synthesis of MIL-68

All the chemicals used in the experiments were analytical grade (AR) without additional purification. The MIL-68 (In) sample was synthesized via the modified solvothermal method [31]. Firstly, 6.0 mmol PTA (p-benzenedicarboxylic acid, 166.13 g mol^−1^) was dissolved in 35 mL DMF with stirring until the solution became clear. 5 mmol indium nitrate hydrate (300.83 g mol^−1^) was dissolved in another 35 mL DMF with the ultrasonic treatment of 30 min. Then both DMF solutions were mixed together in a 100 mL Teflon-lined autoclave under continuous stirring for 10 min. After that, the above-mentioned autoclave was tightly sealed in a matched stainless container and treated at 100 °C for 12 h. Finally, the obtained solid products were purified by centrifugation for three times with ethanol as the dissolvent. The remaining white precipitate was MIL-68 powder.

### Synthesis of MIL-68-Air

The MIL-68 powders as the precursor were annealed at 500 °C for 2 h with a heating of 10 °C min^−1^ in air in muffle furnace. The obtained pale-yellow powders were marked as MIL-68-Air.

### Synthesis of MIL-68-N_2_

The MIL-68 powers as the precursor were firstly loaded in an alumina crucible accommodated by a tube furnace. After that, the precursor was calcined at 600 °C for 2 h with a heating rate of 5 °C min^−1^. Finally, the remained products in tube furnace were cooled to room temperature without any external operation. The residual pitch-black powders were marked as MIL-68-N_2_. The whole calcination treatment was under the nitrogen atmosphere with a constant 30 mL min^−1^ gas flow rate.

### Material Characterization

Scanning electron microscope (SEM) measurements with energy dispersive spectrometer (EDS) studies were carried out on a field emission SEM (ZEISS SUPRA®55). Transmission electron microscopy (TEM) images, high-resolution TEM (HR-TEM) images and electron diffraction patterns were measured with a JEOL JEM-3200FS. X-ray diffraction (XRD) data was collected on a diffractometer (D8 Advance, Bruker) with Cu Kα radiation (2θ ranging from 5° to 80°, λ = 1.541 Å, step size = 0.02°). X-ray photoelectron spectroscopy (XPS) studies were carried out on ESCALAB 250Xi (Thermo Fisher). Spectra were analyzed using XPSPEAK software, and the C1s peak for adventitious hydrocarbons at 284.8 eV was used for binding energy calibration. Electron paramagnetic resonance (EPR) spectra were performed on Magnettech MS 5000 to identify the electrons trapped on oxygen vacancies. The testing parameters were as follows: microwave frequency of 9.44 GHz, microwave power at 10 mW, sweep time of 120 s, magnetic field from 30 to 650 mT, modulation at 0.2 mT, and modulation frequency at 100 kHz.

### Electrode Preparation

3 mg catalyst powders, 1.5 mg carbon black, and 30 μL 5 wt.% Nafion solution (Sigma-Aldrich) were firstly dispersed in the mixed solution of 285 μL ethanol and 285 μL deionized water. Then the suspension was sonicated for 30 min to obtain a homogeneous black ink. After that, the catalyst ink was dropwise onto a carbon paper with the area of 3 × 1 cm^2^ to achieve the loading of 1 mg cm^−2^ as the working electrode: gas diffusion electrode (GDE).

### Electrochemical Measurements

Electrochemical measurements were conducted in a flow cell reactor and measured by the electrochemical workstation of CHI760E. The reactor comprised three compartments or chambers, which could be labeled as gas chamber, cathodic chamber, and anodic chamber. Both the cathodic chamber and the anodic chamber connected with a polytetrafluoroethylene (PTFE) cell outside the flow cell reactor. These two PTFE cells were used to hold the electrolytes. The carbon paper loaded with catalysts worked as the cathode, and on the contrary, the commercial iridium-plated titanium sheet was used as the anode. The Hg/HgO reference electrode was anchored to the case of the reactor. All these electrodes were immersed into the electrolyte of 1 M KOH to guarantee an alkaline environment for target redox reactions at their surfaces. Two pumps provided the power to get the electrolytes flowing, of which the peristaltic pump maintained a speed of 15 r min^−1^ for the electrolyte in the catholyte chamber. The gas chamber and cathodic chamber were separated by the as-prepared GDE, and the catalyst side faced the cathodic chamber. As the reactant, the high-purity CO_2_ gas was compressed into the gas chamber at a flow rate of 20 mL min^−1^. Under this gas pressure, CO_2_ could spread to the other side of the GDE. That formed a three-phase (solid phase: catalyst; liquid phase: catholyte; gas phase: CO_2_) interface, where the cathodic reactions happened. The cathodic chamber and anodic chamber were separated by an anion-exchange membrane (FUMATECH, FAA-3-PK-130). All the potentials in this paper were calibrated to versus RHE without *iR* corrections according to the following equations: *E*_RHE_ = *E*_Hg/HgO_ + 0.059 pH + *E*^0^_Hg/HgO_, where *E*_Hg/HgO_ is the potential difference measured between the working electrode and the reference electrode, pH is the pH of the electrolyte solution, and *E*_RHE_ is the calibrated potential.

The cyclic voltammetry (CV) measurements were performed at a scan rate of 50 mV s^−1^ for 20 cycles to obtain a stable CV curve before other electrochemical measurements. The linear sweep voltammetry (LSV) measurements were carried out 5 times at a scan rate of 10 mV s^−1^ to obtain a stable polarization curve. Electrochemical impedance spectroscopy (EIS) analysis were conducted at − 0.4 V versus RHE at DC potential of 5 mV with the frequency ranging from 100 kHz to 0.03 Hz. CV was used to determine the electrochemical active surface areas (ECSAs) of the samples presented in this paper. The potential was swept in a range from 0.05 V above the open-circuit potential to 0.05 V below the open-circuit potential (OCP) with five different scan rates: 5, 10, 20, 30, and 40 mV s^−1^. All experiments were performed at room temperature.

### CO_2_ Reduction Performance Measurements

In order to evaluate the CO_2_ reduction performances of the catalysts, stepped potentials (range of -0.2 to -1.2, 0.2 V as the interval) were applied to the flow cell reactor. The gas products were quantitatively analyzed by gas chromatography (GC, SHIMADZU GC-2014). A thermal conductivity detector (TCD) was used to quantify hydrogen (H_2_) and oxygen (O_2_), two flame ionization detectors (FID) were used to quantify methane (CH_4_)/carbon monoxide (CO), and ethane (C_2_H_6_)/ethylene (C_2_H_4_)/ethyne (C_2_H_2_), respectively. The reactors were connected with an online GC with a mass flowmeter at the outlet line. This flowmeter could precisely measure the rate of the gas flow from the reactor into the GC. The reading rate would be used to calculate the faradaic efficiencies of the gas phase products. Before the GC started to test, each potential should be kept running for at least 15 min to discharge the residual gas in the equipment and replaced with the fresh gas at the current working potential. The Faradaic efficiency (FE) of gas phase products was calculated as follows:

1 $$\begin{aligned} FE & = \frac{{Q_{g} }}{{Q_{t} }} = \frac{{N \times x_{g} \times e}}{{\int {I{\text{d}}t} }} \approx \frac{{n_{g} \times N_{A} \times x_{g} \times e}}{{\overline{I} \times \Delta t}} \\ & = \frac{{\frac{{ppm \times v_{0} }}{{10^{6} \times Vt}} \times F \times x_{g} }}{{\overline{I} \times \frac{{v_{0} }}{sccm}}} = \frac{{ppm \times F \times x_{g} \times sccm}}{{10^{6} \times V_{t} \times \overline{I}}} \\ \end{aligned}$$

where *Q*_***g***_ and *Q*_*t*_ are the charge transferred to the gas phase products, and the total charge across the catalysts, respectively; *I* is the absolute value of the operating current, *t* is the integral interval of a certain time span; *N* is the total number of calculated gas product molecules; *x*_*g*_ is the number of electrons required by one product molecule in the reduction process, for H_2_ and CO, the *x*_*g*_ is 2; *N*_*A*_ is the Avogadro constant; *e* is the charge of a single electron; *n*_*g*_ is the amount of substance of the gas products; *ppm* (parts per million) is the gas product concentrations listed by GC; *v*_*0*_ is the gas volume inhale by the GC; *F* is the Faradaic constant; *V*_*t*_ is the molar volume of the gas products at the laboratory temperature; *sccm* (standard-state cubic centimeter per minute) is the gas flow rate displayed on the mass flowmeter between the GC and the reactors**;** it should not be the original flow rate which was set as 20 sccm on the upstream of the reactors. Because of the gap between the time when the reaction happens in the reactors and the time the reacted gas flows into the GC, the current should be adopted as $\overline{I}$ which is the current value at the time of 300 s before the GC starts to inhale the CO_2_ gas. Besides, *Δt* is the time taken by the GC to inhale 1 mL of CO_2_ gas. All the units adopt the international system of units.

The liquid products were measured by nuclear magnetic resonance (NMR) spectroscopy. For preparing NMR samples of liquid products, the electrocatalysis was kept at a constant potential for 60 min to ensure a strong NMR signal at higher product concentrations. The NMR samples were prepared by mixing 0.1 mL of the collected electrolyte solution, 0.1 mL of internal standard solution (containing 0.02 μL of dimethyl sulfoxide (DMSO) as internal standard) and 0.4 mL of D_2_O as deuterated solvent. Once sealed, the NMR tube should be shaken for a while to obtain a uniform solution. ^1^H NMR spectra of freshly acquired samples were collected on BRUKER AVANCE III (400 MHz) in water depression mode. The data were analyzed by the MestReNova software. In order to eliminate integration errors, the obtained spectra were firstly calibrated by manual phase corrections and multipoint baseline corrections. The FE of liquid phase products was calculated as follows:

2 $$\begin{aligned} FE & = \frac{{Q_{l} }}{{Q_{t} }} = \frac{{N \times x_{l} \times e}}{{\int {I{\text{d}}t} }} = \frac{{n_{l} \times \frac{{V_{e} }}{{v_{e} }} \times N_{A} \times x_{l} \times e}}{{\int {I{\text{d}}t} }} = \frac{{n_{i} \times \frac{{S_{l} }}{{S_{i} }} \times \frac{{m_{i} }}{{m_{l} }} \times \frac{{V_{e} }}{{v_{e} }} \times F \times x_{l} }}{{\int {I{\text{d}}t} }} \\ & = \frac{{\frac{{\rho_{i} \times v_{i} }}{{M_{i} }} \times \frac{{S_{l} }}{{S_{i} }} \times \frac{{m_{i} }}{{m_{l} }} \times \frac{{V_{e} }}{{v_{e} }} \times F \times x_{l} }}{{\int {I{\text{d}}t} }} = \frac{{\rho_{i} \times v_{d} \times S_{l} \times m_{i} \times V_{e} \times F \times x_{l} }}{{M_{i} \times S_{i} \times m_{l} \times v_{e} \times \int {I{\text{d}}t} }} \\ \end{aligned}$$

where *Q*_*l*_ and *Q*_*t*_ are the charge transferred to the liquid products, and the total charge across the catalysts, respectively; *I* is the absolute value of the operating current, *t* is the integral interval of a certain time span; *N* is the total number of calculated liquid product molecules; *x*_*l*_ is the number of electrons required by one product molecule in the reduction process, for HCOOH, the *x*_*l*_ is 2; *e* is the charge of a single electron; *n*_*l*_ is the amount of substance of the target products; *V*_*e*_ is the volume of all the electrolytes for each test, including catholyte and anolyte; *v*_*e*_ is the volume of the electrolyte held by the corresponding NMR tube; *N*_*A*_ is the Avogadro constant; $\frac{{S_{l} }}{{S_{i} }}$ is the ratio of the areas of the liquid product peaks to the area of the internal standard peak; $\frac{{m_{i} }}{{m_{l} }}$ is the ratio of the numbers of the certain protons in the internal standard and liquid product molecules, for DMSO, the *m*_*i*_ values 6 from two methyl groups, and for HCOOH, *m*_*l*_ values 1 from HCOO^−^; F is Faradaic constant; *ρ*_*i*_ is the density of internal standard, *v*_*i*_ is the volume of the internal standard solution held by the corresponding NMR tube; *M*_*i*_ is the molar mass of the internal standard, for DMSO, it is 78.13 g mol^−1^. Unlike the $\int {I{\text{d}}t}$ for gas products, the $\int \mathrm{I dt}$ which covers a quantitative amount of time for the valuation of liquid products can be calculated exactly because the NMR is not a real-time test. All the units adopt the international system of units.

### CO_2_ Reduction Stability Measurements

The catalyst stability was also assessed with flow cell reactor. Different from the CO_2_ reduction measurements, the peristaltic pump speed was set at 10 r min^−1^ instead of 15 r min^−1^ to reduce the electrolyte pressure in the catholyte chamber that could maintain a good stability against electrolyte flooding penetration and formate crossover. Firstly, 4 times of cyclic voltammetry ranging from + 0.8 to − 0.6 V (versus RHE, scan rate at 0.05 V s^−1^) and 5 times of linear sweep voltammetry ranging from + 0.15 to − 0.7 V (versus RHE, scan rate at 0.01 V s^−1^) were performed to activate and stabilize the catalysts. After that, the current density was kept constantly at − 100 mA during the whole test. And at the same time, the GC gave the real-time testing results of the gas products. To estimate the catalyst stability on liquid products, the catholyte and anolyte were taken out of the PTFE cells every 12 h and at the same time another 50 mL & 50 mL of fresh solutions were poured into the two cells. This replacement process should be as quick as possible to lessen impacts of replacement on the stability test. For NMR analyses, the sample were also prepared by mixing 0.1 mL of the collected electrolyte solution, 0.1 mL of internal standard solution and 0.4 mL of D_2_O. But it was worth noting that, the internal standard solution transferred into the NMR tube by a pipette contained 0.2 μL of DMSO, which was 10 times of the amount as that in the above electrochemical measurements. It was because the reaction time scale of each NMR sample from stability tests and above electrochemical measurements was 12 and 1 h, respectively.

### Ex situ XAFS Measurements

The X-ray absorption fine structure (XAFS) data for the as-prepared MIL-68, MIL-68-Air, and MIL-68-N_2_ samples were collected on the Beamline 20-BM facility of the Advanced Photon Source (APS) at Argonne National Laboratory (ANL). Energies were selected using a double-crystal Si (111) monochromator while the detection *I*_0_ and *I*_T_ used standard ionization chambers. To calibrate the energy, a reference In metal foil was used, which was measured in line with the samples. All XAFS temperature in transmission mode with continuous scanning between 27,700 and 28,860 eV and a commercial In_2_O_3_ (Aladdin, 99.99% metal basis) was also measured as a reference to calculate the coordination number and valence state.

### H-Cell Measurements

In order to find the suitable testing potentials for the operando XAFS measurement, a H-cell reactor was employed, because it could provide the similar reaction conditions as the in situ electrochemical cell reactor did. The H-cell reactor comprised two chambers separated by a proton-exchange membrane (DUPONT, N115). The 0.5 M KHCO_3_ solution pre-saturated with CO_2_ was used as the electrolyte, and Ag/AgCl electrode and platinum were applied as the reference electrode and counter electrode, respectively. The carbon paper loaded with catalyst was fixed by a glassy carbon electrode holder as the working electrode. The back side of the carbon paper without catalyst loading was sealed by waterproof tape. The electrolyte was inflated by a high-purity CO_2_ (99.999%) gas flow at 20 SCCM for 30 min to obtain a CO_2_-saturated solution. And CO_2_ was continuously bubbled into the electrolyte at the same flow rate throughout the power-on state. The stepped potentials of − 0.445, − 0.645, − 0.845, − 1.045, − 1.245, and − 1.445 V versus RHE were applied at the three-electrode system. And the potentials were held for 5, 1, 1, 1, 1, and 1 h respectively to make sure that liquid product concentrations were at high level to be detected by NMR. The gas phase product analyses were carried out by online GC.

### Operando XAFS Measurements

The electronic and local structures of MIL-68-N_2_ catalyst under operating conditions were characterized on the Beamline 12-BM-B at APS/ANL. Operando XAFS measurements were performed in fluorescence mode over a custom-made electrochemical cell containing a catalyst-coated carbon paper sheet as the working electrode, a platinum wire counter electrode and an Ag/AgCl reference electrode. After filling the electrochemical cell with 0.5 M KHCO_3_ aqueous solution, purged it with flowing CO_2_ for 30 min to obtain a CO_2_-saturated solution, and CO_2_ was continuously bubbled into the electrolyte throughout the power-on state. Then the first In K-edge XAFS spectrum were collected and dominated as “fresh”. The activation of catalysis was performed with cyclic voltammetry scanning for 5 cycles with a scan rate of 0.05 V s^−1^. Another XAFS spectrum was collected afterward, which was named as “CVs”. After this, the operando XAFS spectra were collected when applied with reduction potentials of − 0.445, − 0.845, − 1.045, − 1.245, and − 1.445 V versus RHE, sequentially. The duration time for each potential was 1 h. In foil and In_2_O_3_ were used as the references to calculate the valence states and coordination numbers. All the XAFS data analysis were undertaken using the Demeter software package [32]. XAFS spectra were calibrated, background subtracted, and normalized, then the XAFS oscillations were extracted using a similar k-range of ≈3.0–12.2 Å^−1^ for all In-K edge spectra and k^2^ weighted data for the Fourier transform. The coordination numbers (CN) and interatomic distances (R) were estimated by curve-fitting analysis of the XAFS data in real space.

### DFT Calculations

To study the electronic structures of In_2_O_3-x_ and energetic trends of CO_2_RR, DFT calculations based on the CASTEP packages have been applied in this work [33]. In this work, we selected the generalized gradient approximation (GGA) and Perdew–Burke–Ernzerhof (PBE) to supply accurate descriptions for the exchange–correlation interactions [34–36]. Based on the ultrasoft pseudopotentials, the plane-wave basis cutoff energy has been set to 380 for the geometry optimizations. The Broyden–Fletcher–Goldfarb–Shannon (BFGS) algorithm has been used for energy minimizations [37]. The coarse quality of k-points has been selected for the geometry optimizations based on the balance between calculation efficiency and accuracy. For all the geometry optimizations, we have set the following convergence criteria that Hellmann–Feynman forces should not exceed 0.001 eV Å^−1^, the total energy difference should be less than 5 × 10^–5^ eV atom^−1^, and the inter-ionic displacement should be smaller than 0.005 Å.

## Results and Discussion

### Synthesis and Characterizations of In_2_O_3-x_@C Nanocorn

**Fig. 1:**
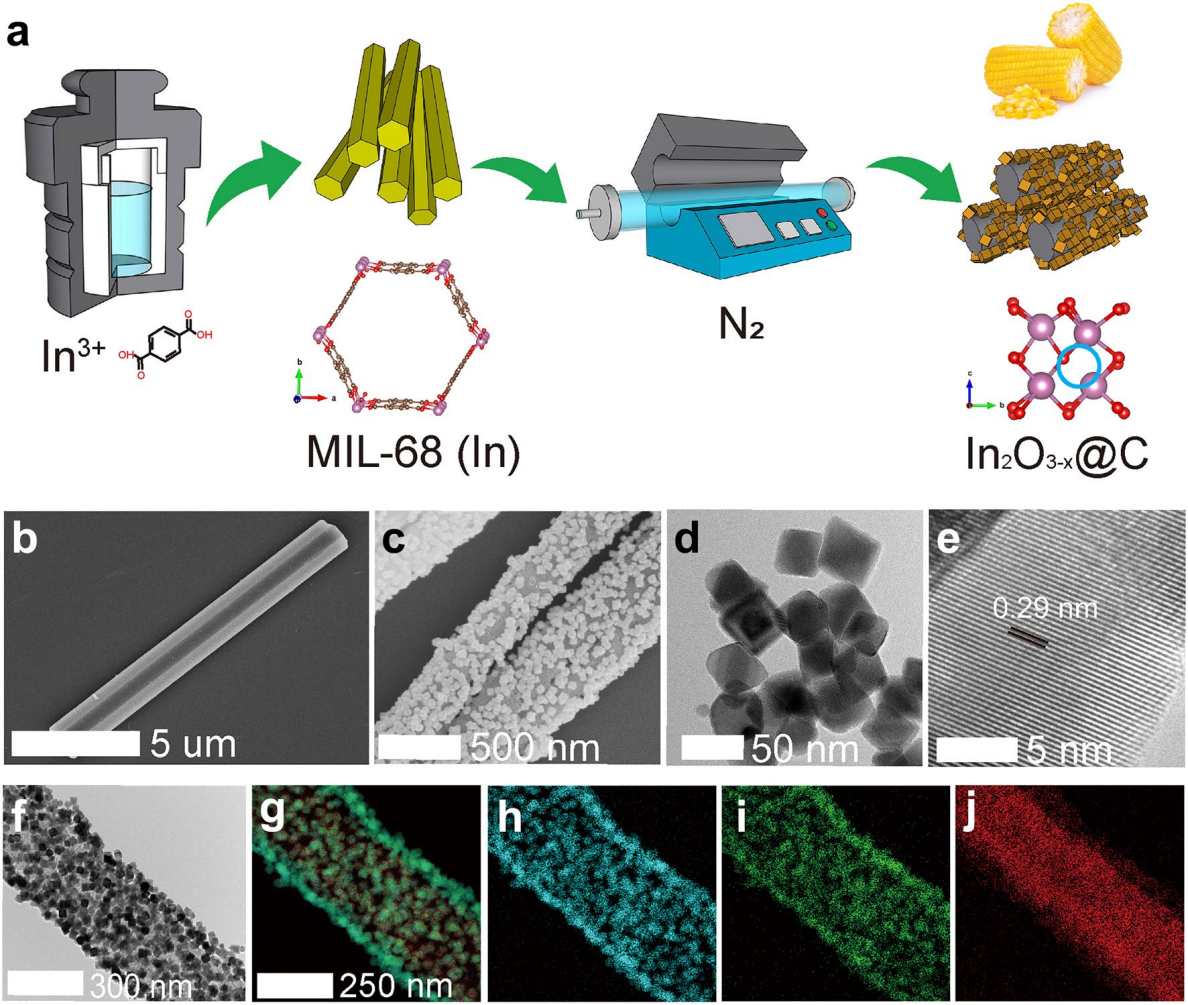
**a** Schematic illustration of the synthesis process. **b–c** SEM images of **b** MIL-68 (In) hexagonal nanorod and **c** MIL-68-N_2_ nanocorn. **d** TEM and **e** high-resolution TEM (HR-TEM) images of the nanocubes in the MIL-68-N_2_ sample. **f** TEM image and **g–j** the corresponding element mapping images of a single nanocube decorated nanorod on **g** all detected elements, **h** In element, **i** O element and **j** C element. The nanocubes in **d** were obtained from MIL-68-N_2_ nanocorn through sonication

The overall synthesis of the nanocorns is illustrated in Fig. 1a. MIL-68 (In MOF) was first synthesized through a modified solvothermal method (details in the experimental section in SI), followed by annealing transformation in N_2_ atmosphere. The formation of the MIL-68 (In) nanorod template is ascertained by their typical XRD peaks as shown in Fig. S1. Figures 1b and S2 show the hexagonal nanorod morphology of the pre-formed MIL-68, which is consistent with the previously reported results [31, 38]. For comparison with MIL-68-N_2_, we also prepared MIL-68-Air through the heat treatment of MIL-68 in air instead of N_2_ (Fig. 1a). It can be seen from Fig. S3 that the smooth surface of MIL-68 became rough after the transformation to MIL-68-Air, which remained the nanorod shape. The SEM image of MIL-68-N_2_ in Fig. 1c and the corresponding TEM image in Fig. 1f show that the overall morphology remained nanorod-like but more accurately it is now like a nanocorn with cubic shape nanoparticles densely and uniformly dispersed on the surface of a carbon cob. Figure 1d-e shows TEM and HR-TEM images of the surface nanoparticles from the MIL-68-N_2_ sample, which clearly portray the cubic shape of the nanoparticles (Fig. 1d). The lattice fringes with the *d*-spacing of 0.29 nm corresponding to the plane of (222) of In_2_O_3_ (Fig. 1e). The elemental mapping images of MIL-68-N_2_ shown in Fig. 1g–j reveal the distinctive core@shell morphology of the nanocorn: In and O are distributed uniformly in the nanocube shell of the nanocorn, while carbon is distributed uniformly enclosed in the core as the cob of the nanocorn. To further confirm this inference, we removed the surface nanoparticles from the nanocorns through sonication, and the EDS mapping images and the HR-TEM of the sonicated nanocorns are shown in Figs. S4 and S5a, respectively. Only the signal of carbon element was detected (Fig. S4), and no crystal lattice was observed (Fig. S5a), indicating the amorphous nature of the carbon cob of the nanocorns, which is also consistent with the Raman spectrum of MIL-68-N_2_ shown in Fig. S5b. These results confirmed that we have established that the MIL-68-N_2_ sample is corn-like nanostructure with indium oxide nanocubes densely and uniformly anchored as a shell on amorphous carbon nanorod as the cob (In_2_O_3-*x*_@C).

**Fig. 2:**
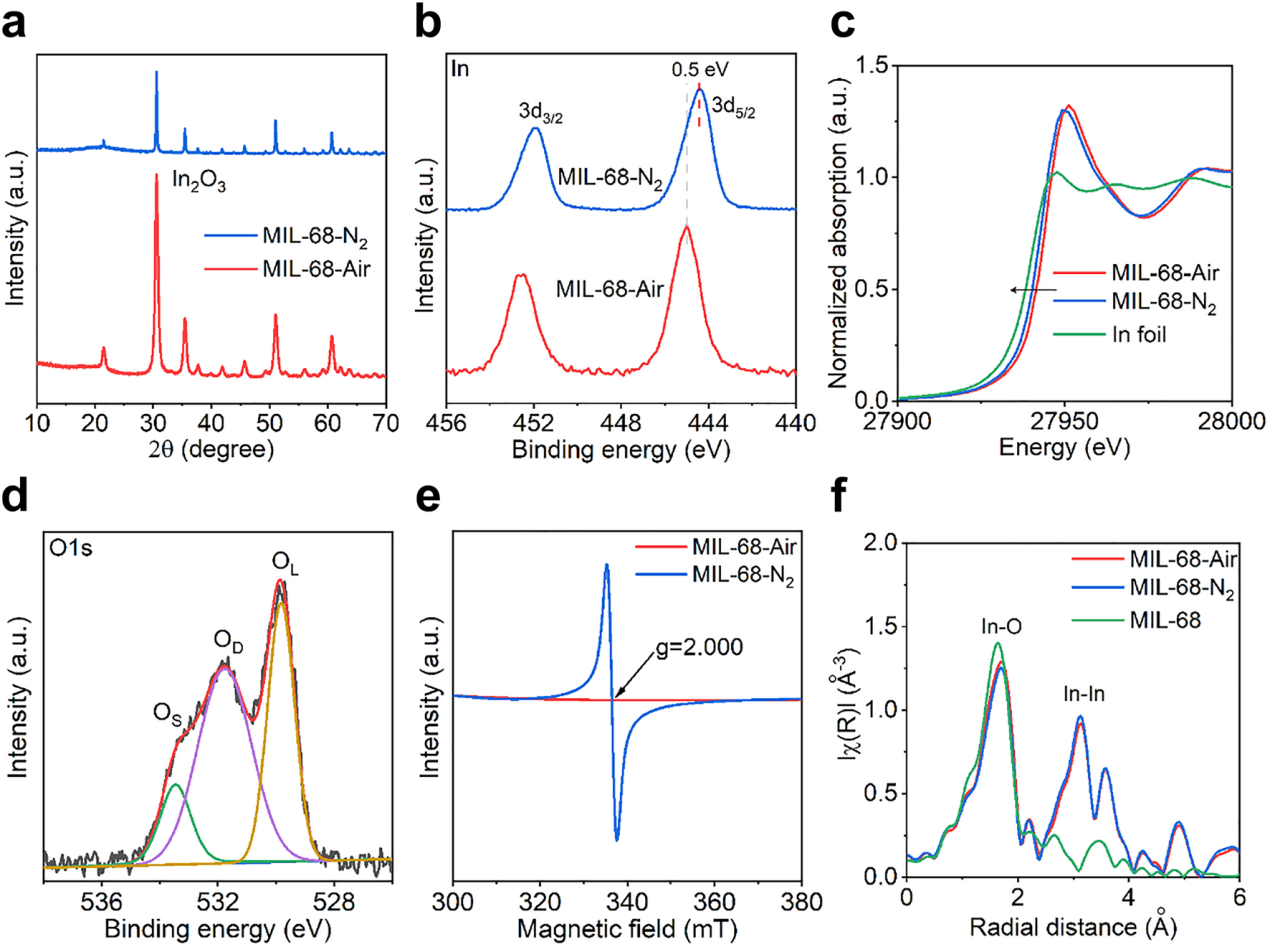
**a** XRD patterns, **b** In 3*d* XPS spectra and **c** In K-edge XANES spectra of the different samples. **d** O 1*s* XPS spectrum of MIL-68-N_2_. **e** EPR spectra of MIL-68-Air and MIL-68-N_2_. **f** Fourier-transformed (FT) *k*^*3*^-weighted R space EXAFS spectra of the different samples

The XRD patterns of MIL-68-Air and MIL-68-N_2_ are shown in Fig. 2a. Both samples only show the diffraction peaks of In_2_O_3_ (PDF#06–0416), but the diffraction peak intensity of MIL-68-N_2_ is generally lower than that of MIL-68-Air due probably to the omnipresent amorphous carbon nanorods shelled by the In_2_O_3_ nanocubes in MIL-68-N_2_ [39]. Figure 2b–c shows the XPS and XAFS spectra of MIL-68-Air and MIL-68-N_2_, respectively, focusing on the valence state of In. As shown in Fig. 2b, the two peaks of MIL-68-Air located at 445 and 452.6 eV are assigned to In 3*d*_5/2_ and In 3*d*_3/2_ in In_2_O_3_. In comparison, the corresponding two peaks in MIL-68-N_2_ are shifted by about 0.5 eV to lower binding energies, indicating a lower valence state of the surface In in MIL-68-N_2_ [14, 17]. Figure 2c shows normalized In K-edge X-ray absorption near-edge structure (XANES) spectra of the samples and for comparison, the In foil. The In K-edge of MIL-68-N_2_ is shifted toward lower energy from that of MIL-68-Air, suggesting a lower average In valence state [25, 26]. This finding is consistent with the XPS result.

To understand the origin for the lower In valence state in MIL-68-N_2_, O 1*s* XPS spectrum of MIL-68-N_2_ was performed and shown in Fig. 2d. The O 1*s* band was composed of three peaks that were labeled as O_L_ (lattice oxygen), O_D_ (oxygen vacancy), and O_S_ (surface adsorbed oxygen). The large area of O_D_ in O 1*s* indicated abundant oxygen vacancies in MIL-68-N_2_ [40]. The EPR spectrum of MIL-68-N_2_ in Fig. 2e shows a strong signal at g = 2.000, which was ascribed to the electrons trapped around the oxygen vacancies. For MIL-68-Air, nearly no oxygen vacancy could be detected from the XPS and EPR results as shown in Figs. S6 and 2e. Figure 2f displays the Fourier transform of the extended EXAFS with *k*^*3*^ weighting. The peaks between 1  and  2 Å in *R* space are regarded as the first In-O shell, while the peaks located between 2.5  and  4 Å are associated with the In-In shell [25]. The peak position and intensity qualitatively revealed distinct local atomic arrangements for the samples of MIL-68, MIL-68-Air, and MIL-68-N_2_. In the hexagonal MOF nanorod of MIL-68, since most of the In atoms were coordinated with O in large organic molecules, the In-O shell dominates the local electronic structures and thus appears as one main peak in the *R* space. After heating treatment, the In-In shell appeared due to the formation of the cubic In_2_O_3_ phase. However, the samples annealed in air and N_2_ showed different intensity ratios of In-O peak to In-In peak. A small value of *I*_In-O_/*I*_In-In_ shall indicates O vacancy in the local atomic structure since there would be less O in the first In-O shell. Comparatively, the MIL-68-N_2_ has a distinctly smaller *I*_In-O_/*I*_In-In_ value (1.296) than MIL-68-Air (1.400), suggesting abundant O vacancies have been created in the In_2_O_3_ nanocubes. These results point to a scenario that the lower In valence state in MIL-68-N_2_ was caused by the abundant oxygen vacancies. With the ability to promote the adsorption and activation of CO_2_ as reported previously, such oxygen vacancies could benefit electrochemical CO_2_ reduction [15, 25].

### CO_2_ Electroreduction Performance

**Fig. 3:**
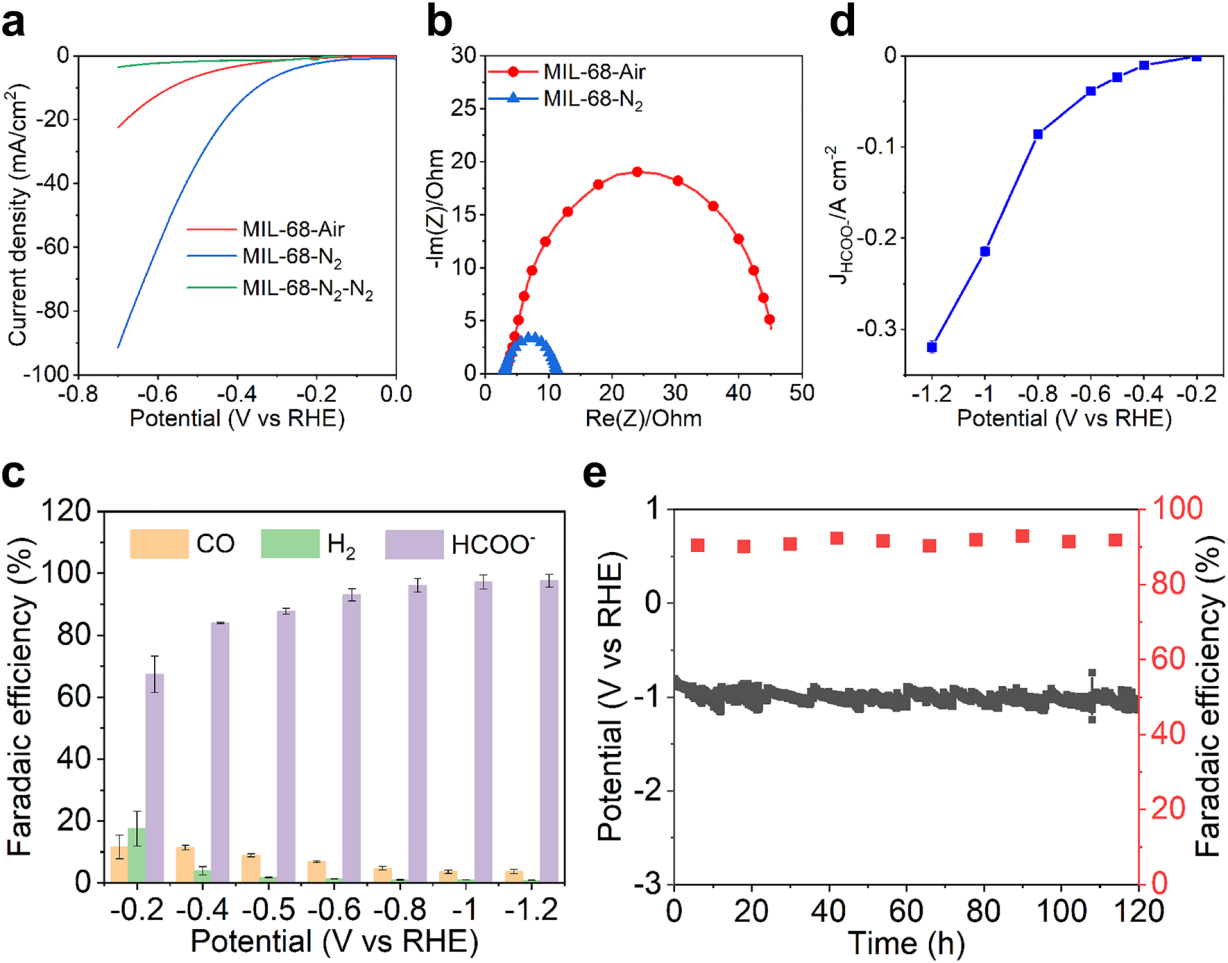
**a** Polarization curves at a scan rate of 10 mV s^−1^. MIL-68-N_2_-N_2_ refers to testing the MIL-68-N_2_ sample in N_2_ instead of CO_2_ as a control experiment. **b** Nyquist plots obtained at -0.4 V vs. RHE. **c** Product distributions in terms of FE, and **d** current density of formate vs. the applied potentials for the MIL-68-N_2_ catalyst. **e** Long-term durability of the MIL-68-N_2_ catalyst at the current density of 100 mA cm^−2^ for more than 120 h. Every 12 h, the old electrolyte was taken out for NMR measurement, and the fresh electrolyte was supplied quickly. All the data reported here are without *iR* correction

The electrochemical CO_2_ reduction reaction of MIL-68-Air and MIL-68-N_2_ was performed in 1 M KOH electrolyte in a flow cell system. The LSV polarization curves of the catalysts are shown in Fig. 3a. Remarkably, the current density of MIL-68-N_2_ is much higher than that of MIL-68-Air. As a control experiment, the MIL-68-N_2_ catalyst was also tested in N_2_ instead of CO_2_ and thus labeled as MIL-68-N_2_-N_2_ as shown in Fig. 3a. The neglectable current density of the control sample is strong evidence that the current density of MIL-68-N_2_ was indeed from CO_2_RR. The ECSAs of MIL-68-Air and MIL-68-N_2_ were estimated by measuring the double-layer capacitance as shown in Fig. S7. The similar ECSAs suggest that the performance difference of MIL-68-Air and MIL-68-N_2_ originates from their different intrinsic activities. The Nyquist plots collected at − 0.4 V versus RHE are shown in Fig. 3b. The much smaller radius of the semi-circle is indicative of a much lower charge transfer resistance of MIL-68-N_2_ than that of MIL-68-Air. The combination of abundant oxygen vacancies in In_2_O_3-*x*_ and the amorphous carbon cob have contributed to the improved charge transfer conductivity, giving rise to the much large current density in MIL-68-N_2_.

Figure 3c shows the CO_2_RR product distributions (FEs) versus the applied potentials from − 0.2 to − 1.2 V versus RHE over MIL-68-N_2_. Gaseous and liquid products were quantified by GC and NMR, respectively, and only HCOOH in the liquid products were detected as shown in Fig. S8. At a very low potential of − 0.4 V, the FE of HCOOH reached 84%, which is to be compared with the FEs of other In-based or Bi-based catalysts reported in the literature (below 80% at − 0.5 V) [13–17, 19, 21, 30, 41–46]. When the potentials increased to − 0.8 V, the FE of HCOOH went up further to beyond 96%. The partial current density of HCOOH for MIL-68-N_2_ at different potential is shown in Fig. 3d. The *J*_HCOOH_ is − 215 mA cm^−2^ at − 1.0 V versus RHE without *iR* correction, much higher than those reported previously as summarized in Table S1. Furthermore, the long-term durability of the MIL-68-N_2_ catalyst was performed, and the results are shown in Figs. 3e and S9. At the current density of 100 mA cm^−2^, the applied potential remained stable for more than 120 h, and the FE for HCOOH kept above 90% during the whole reaction process. The FEs of HCOOH at large current densities were also measured to investigate the potential practical application of MIL-68-N_2_ in a flow cell. As shown in Fig. S10, the FE of HCOOH remained at about 88% even for a current density as large as 1000 mA cm^−2^.

### Density Functional Theory Calculations

**Fig. 4:**
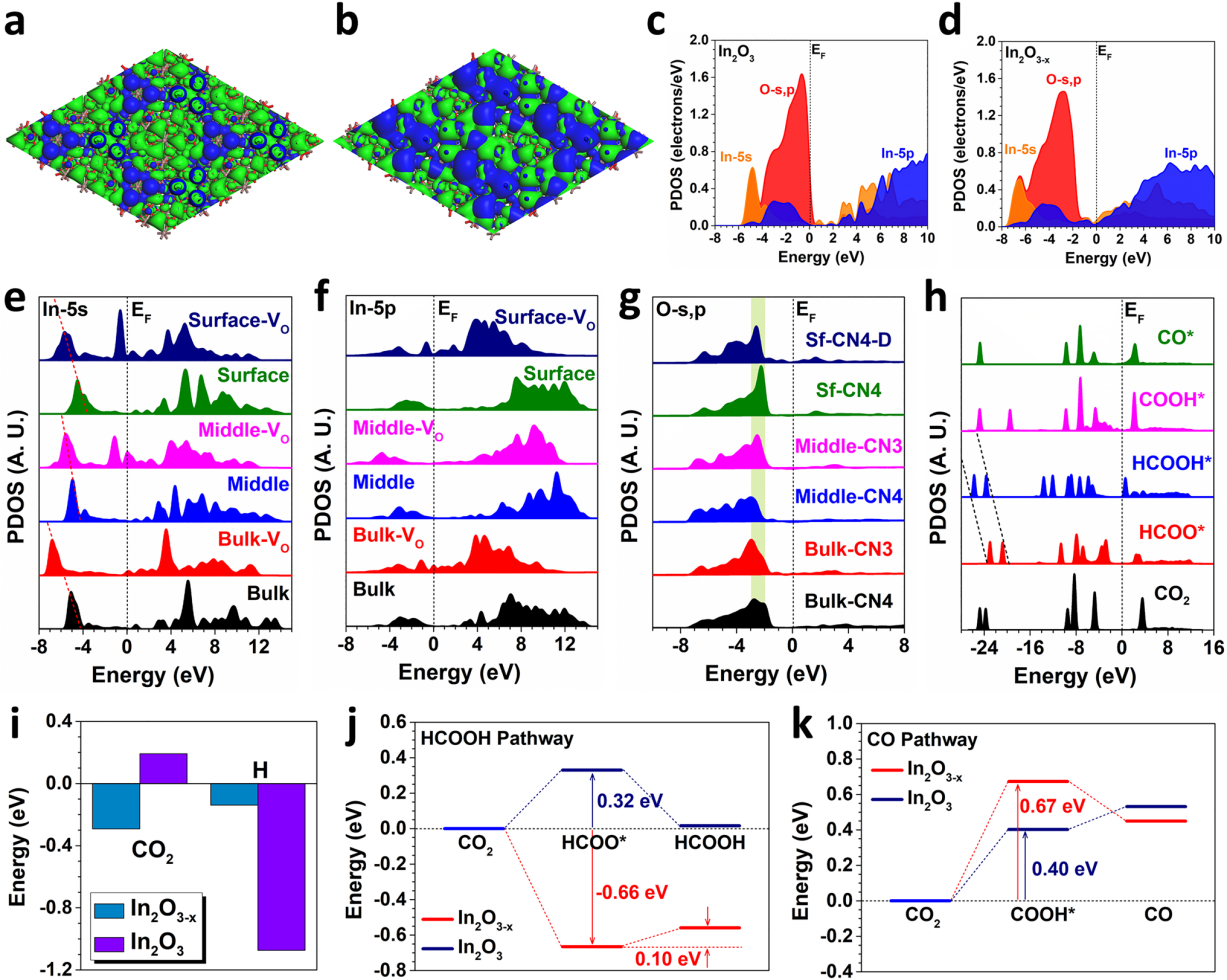
The 3D contour plot of electronic distribution near Fermi level of **a** In_2_O_3_ and **b** In_2_O_3_ with O vacancy. Brown balls = In and red balls = O. Blue isosurface = bonding orbitals and green isosurface = anti-bonding orbitals. The PDOS of **c** In_2_O_3_ and **d** In_2_O_3_ with O vacancy. **e** The site-dependent PDOS of In-5*s* in In_2_O_3-*x*_. **f** The site-dependent PDOS of In-5*p* in In_2_O_3-*x*_. **g** The site-dependent PDOS of O-*s*,*p* in In_2_O_3-*x*_. **h** The PDOS of key adsorbates in In_2_O_3-*x*_. **i** The binding energy comparisons. **j** The reaction energy of HCOOH formation in In_2_O_3_ and In_2_O_3-*x*_. **k** The reaction energy of CO formation in In_2_O_3_ and In_2_O_3-*x*_

To understand the CO_2_RR performances in In_2_O_3-*x*_, we have applied DFT calculations for electronic structures and CO_2_RR reaction trends. For the pristine In_2_O_3_ without oxygen vacancies, we notice that the bonding orbital and anti-bonding orbitals near the Fermi level (E_F_) are evidently separated, leading to the energy barriers of electron transfer (Fig. 4a). The surface oxygen shows evident distortion, where part of the protruding oxygen atoms has formed a triangle active region with a concentration of orbital bonding. After the oxygen vacancies are introduced, the surface electronic structures have been perturbed (Fig. 4b). The electroactivity of surface exposed In sites has been activated, which results in larger electroactive regions to promote the CO_2_RR. The improved coupling between the bonding and anti-bonding orbitals leads to the enhanced electron transfer from the surface toward the adsorbates. More in-depth electronic structure comparisons are demonstrated by the projected partial density of states (PDOSs) (Fig. 4c). In In_2_O_3_, the valence band maximum (VBM) is dominated by O-*s*,*p* orbitals while the 5*s*, 5*p* orbitals of In mainly contribute to the conduction band minimum (CBM). We notice an energy gap of 2.50 and 2.82 eV between VBM and CBM based on the weights of PDOS and the orbital level interval, respectively, with the appearance of several small gap states. In contrast, the oxygen vacancies have downshifted the overall orbitals (Fig. 4d). Moreover, both O-*s*,*p*, and In-5*p* contributed to the VBM. The energetic barrier for electron transfer has been significantly alleviated, which promotes the CO_2_RR. The site-dependent PDOS of In-5*s* shows that the neighboring vacancies significantly increase the electron density near *E*_F_ from the bulk to the surface (Fig. 4e). The In-5 s bands are also downshifted, becoming more electron-rich for the reduction. A similar phenomenon is also noted in In-5*p* through the site-dependent PDOS (Fig. 4f). These results further reveal that the introduction of vacancies is able to activate the local In sites to contribute to the CO_2_RR. Meanwhile, the O-*s*,*p* orbitals show nearly unchanged electronic structures for different coordination environments (Fig. 4g). This confirms that the improved electroactivity of In_2_O_3-*x*_ is attributed to the activated In sites by introducing large amounts of oxygen vacancies.

Then, we have further compared the PDOSs of key adsorbates of CO_2_RR to illustrate the product preference, i.e., selectivity (Fig. 4h). The conversion of HCOO* and HCOOH* shows a stronger linear correlation than that of the COOH* and CO*, supporting a smoother electron transfer during CO_2_RR. The investigations of electronic structures have revealed the activation of electrocatalyst by the oxygen vacancies, which is the key factor for the improved CO_2_RR performance. As a competitive reaction, the hydrogen evolution reaction (HER) needs to be suppressed to guarantee the efficient production of HCOOH. Notably, the adsorption of H and CO_2_ shows a strong contrast (Fig. 4i). In In_2_O_3_, the proton adsorption is highly preferred due to the much lower energy, leading to the strongly lowered performances of CO_2_RR. With the abundant surface oxygen vacancies, the CO_2_ adsorption has been improved while the proton adsorption has been largely suppressed, supporting the promotion of the CO_2_RR. For the formation of HCOOH, we notice that In_2_O_3-*x*_ has shown a spontaneous formation trend of HCOO* with an energy drop of − 0.66 eV (Fig. 4j). In comparison, the In_2_O_3_ makes an energy barrier of 0.32 eV, leading to a lower conversion efficiency to HCOOH. For the second hydrogenation step, In_2_O_3-*x*_ exhibits a subtle energy barrier of 0.10 eV. The energetically favorable formation of HCOOH on In_2_O_3-*x*_ supports the superior CO_2_RR performances. For the CO reaction pathway, it is noted that both In_2_O_3-*x*_ and In_2_O_3_ display unfavorable reaction trends (Fig. 4k). The formation of CO is strongly limited by the difficult conversion to COOH* on both In_2_O_3-*x*_ and In_2_O_3_ with an energy barrier of 0.67 and 0.40 eV, respectively. The large energy barriers determine the low CO production as well as the absence of C2+ products, which are consistent with the experimental results. Therefore, the optimized electronic structures of In_2_O_3-*x*_ are critical to realize the high selectivity and efficiency of CO_2_RR.

### Operando XAS Measurements

**Fig. 5:**
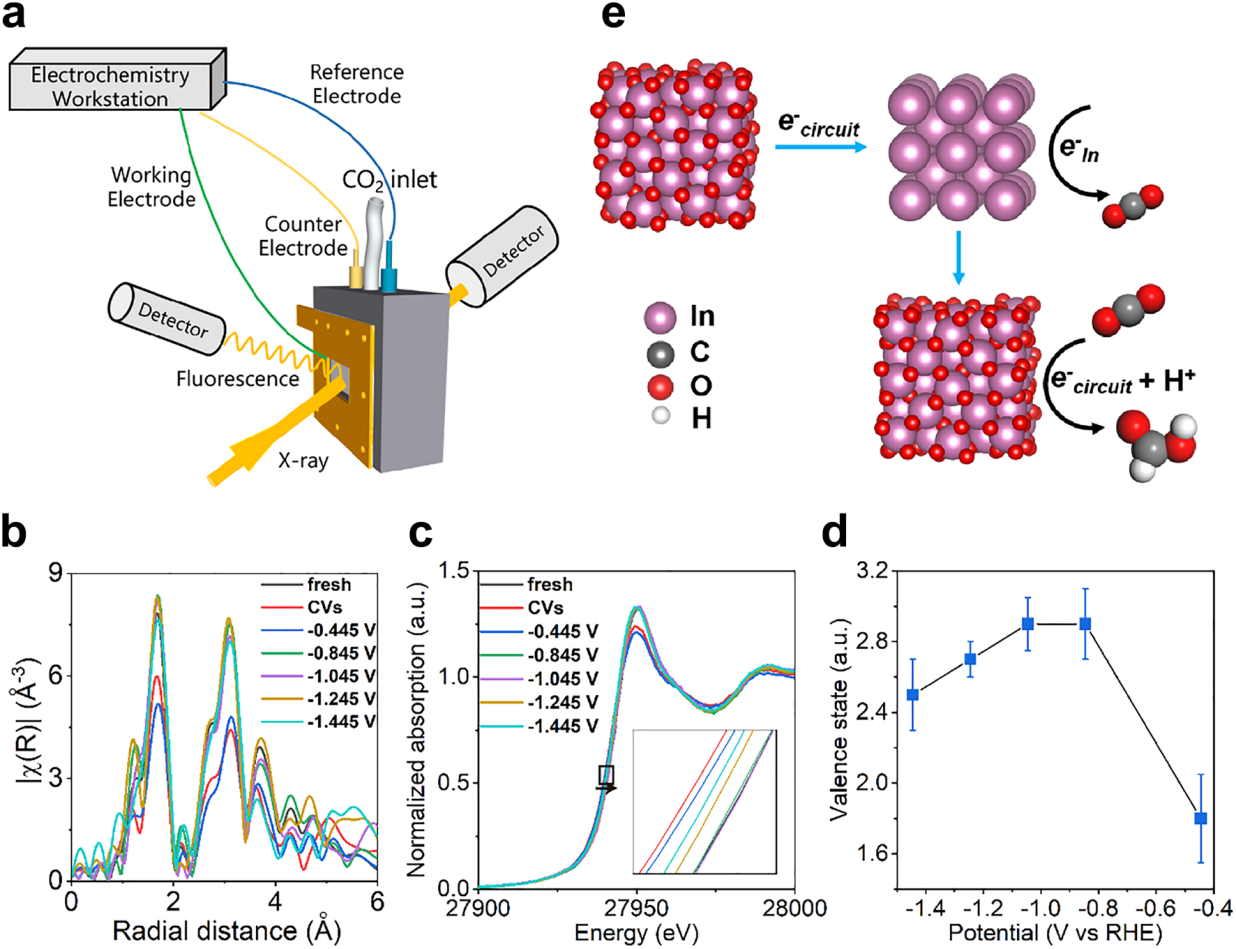
**a** Schematics of the experimental set-up. **b** Fourier-transformed (FT) *k*^3^-weighted R space EXAFS spectra and **c** In K-edge XANES spectra of MIL-68-N_2_ tested under different applied potentials. **d** Changes of In valence state in MIL-68-N_2_ under different applied potentials. **e** The proposed reaction mechanism based on the operando measurements

Clarification of the active sites of a catalyst is essential to understanding its catalytic reaction mechanism. Operando XAS measurements were performed in fluorescence mode over a custom-made electrochemical cell, as shown in Fig. S11, to study the catalytic active sites, and the overall set-up is shown in Fig. 5a. To obtain the suitable CO_2_RR potentials of the MIL-68-N_2_ catalyst for the operando XAFS measurements, the electrochemical CO_2_ reduction reaction on MIL-68-N_2_ was performed in 0.5 M KHCO_3_ aqueous solution in a H-cell system. The CO_2_RR product distributions in terms of FEs versus the applied potentials from − 0.445 to − 1.445 V vs. RHE over MIL-68-N_2_ are presented in Fig. S12. Nearly no HCOOH was generated at − 0.445 V versus RHE, but when the applied potentials were increased to − 0.845 and − 1.045 V versus RHE, the FEs of HCOOH were above 80%. However, the FEs decreased gradually when the applied potentials were further increased to − 1.245 and − 1.445 V versus RHE. Accordingly, we chose − 0.445, − 0.845, − 1.045, − 1.245, and − 1.445 V for the operando XAFS measurements, and the results are shown in Fig. 5b-d. Shown in Fig. 5b are the EXAFS spectra collected for the fresh MIL-68-N_2_ catalyst, the activated catalyst after the CV cycling (0.555 to − 0.845 V vs. RHE) and the operating catalyst at different potentials. The EXAFS data showed an obvious change in the In-O shell when the catalyst was under certain operating conditions, indicating that the catalytic reaction is highly related to the first In-O shell structure. The coordination number (CN) of In was obtained by fitting the first In-O cell of the EXAFS data as shown in Fig. 5b and Table S2. The standard In_2_O_3_ structure features an In-O CN of 6, while the CN is reduced to ~ 4 for MIL-68-N_2_ after CVs. or under the applied potential of − 0.445 V because of the reduction of Indium oxide and the loss of coordinated oxygen. When the applied potential was increased to − 0.845 ~ − 1.045 V, which highly favors the conversion of CO_2_ to HCOOH, the CN was increased to around 5. At these potentials, CO_2_ can adsorb on the O vacancy neighbored In sites through oxygen coordination. In other words, the In sites are the active sites and the coordination atom is oxygen instead of C so that CO_2_RR shall follow the HCOO* channel, in agreement with the DFT results (vide supra).

CV curves of the MIL-68-N_2_ catalyst are shown in Fig. S13a, and an obvious reduction peak of In^3+^ to In was observed at ~ − 0.17 V versus RHE, indicating that the reducing atmosphere during CO_2_ reduction reduces indium oxide to In metal [13, 14]. The XRD pattern (Fig. S13b) and the XANES spectrum (Fig. S13c) of the MIL-68-N_2_ catalyst collected after the electrolysis support this result. However, it is not reasonable to assume that In metal is the active site for the CO_2_RR to HCOOH judging simply from the above experimental observation. Operando XAFS is a powerful tool for obtaining valence information and for tracking how it evolves during catalytic reactions. Figure 5c shows normalized In K-edge XANES spectra of the fresh MIL-68-N_2_ catalyst, MIL-68-N_2_ after the CV cycling and MIL-68-N_2_ at different potentials and in different operating catalytic states. After the CV test, the In K-edge shifted toward lower energy from that of fresh MIL-68-N_2_, suggesting a reduced average In valence state. But the In K-edge shifted very little when a constant potential of − 0.445 V versus RHE was subsequently applied. Surprisingly, when the applied potentials were increased to − 0.845 and − 1.045 V versus RHE, the In K-edge shifted toward higher energy and nearly overlapped that of fresh MIL-68-N_2_. For much higher potentials of − 1.245 and − 1.445 V versus RHE, the In K-edge shifted toward lower energy again. Collectively, the average In valence state was the lowest (metallic In rich) at − 0.445 V, the medium at − 1.245 and − 1.445 V, and the highest (In^3+^ rich) at − 0.845 and − 1.045 V.

The valence states of In in MIL-68-N_2_ at different potentials of CO_2_RR were examined by using the K-edge energy shift with respect to the commercial In_2_O_3_ sample as shown in Fig. 5d. For the negative potential of − 0.445 V, at which nearly no HCOOH production occurred from CO_2_RR, the In valence state was below + 2. When the FE of HCOOH enhanced to above 85% as the set potential was increased to − 1.045 V versus RHE, the valence state of In was about +3. Further increasing the set potential to − 1.445 V versus RHE, the FE of HCOOH decreased to 51% and the valence state of In decreased to ~  + 2.5. Considering the above *in situ* spectroscopic results, we can propose the reaction mechanism as illustrated in Fig. 5e. At the negative potentials, In^3+^ is not stable and will be reduced. However, when the reaction of CO_2_ to HCOOH occurs, the reduced In shall be re-oxidized to In^3+^ via charge transfer to the adsorbed CO_2_ perhaps accompanied by interfacial and surface reconstruction. The In^3+^ species will then act as the catalytic active sites for HCOOH production during the subsequent CO_2_RR cycling process.

## Conclusions

In summary, a fine-grained corn design is demonstrated for a generic indium oxide electrocatalyst toward CO_2_RR obtained by the MIL-68 (In) to MIL-68-N_2_ topotactic transformation via a simple annealing procedure. The oxygen vacancies in In_2_O_3-*x*_ shell and the in situ formed carbon nanorod cob bring in the high activity of In sites and low charge transfer resistance, resulting in the excellent performance for electrochemical reduction of CO_2_ to HCOOH. MIL-68-N_2_ achieves 84% FE with J_HCOOH_ of 11 mA cm^−2^ at a very low potential of − 0.4 V versus RHE, and the FE and J_HCOOH_ can further enhance to 97% and 215 mA cm^−2^ under the potential of − 1.0 V versus RHE. The stable operation time is over 120 h at 100 mA cm^−2^. DFT calculations have unraveled that oxygen vacancies created an electron-rich environment for the In active sites, which not only increases the reducing power at the active sites but also pulls down the energy barrier for electron transfer. This results in the much higher activity of the In sites for the CO_2_RR toward the selective formation of HCOOH. Moreover, the reaction mechanism was carefully studied through operando X-ray absorption spectroscopy under a series of potentials. Because of the applied negative potential, In^3+^ is easily reduced to metallic In at the initial catalyst activation stage, but then rapidly re-oxidized to In^3+^ by CO_2_ via facile electron transfer and subsequently kept at this valence state as the active catalytic site for HCOOH production. By leveraging the new material platform, this work unveils the electronic and coordination reconstruction of the active sites at the initial catalyst activation stage, confirms the active sites for the actual catalytic CO_2_ reduction cycles for the highly selective HCOOH production. This work will guide future efforts to achieve efficient and stable catalysts for industrial CO_2_ reduction processes.

## Supplementary Information

Below is the link to the electronic supplementary material.

Supplementary file1 (PDF 1329 kb)
