# Supplementary material for: MOF-Transformed In2O3-x@C Nanocorn Electrocatalyst for Efficient CO2 Reduction to HCOOH
Source: Nanomicro Lett. 2022 Aug 17;14:167. doi: 10.1007/s40820-022-00913-6 (PMC9385936; doi:10.1007/s40820-022-00913-6)
Supplement: Supplementary file 1 — Supplementary file1 (PDF 1329 kb) [file 40820_2022_913_MOESM1_ESM.pdf]

Supporting Information for

MOF Transformed In_2O_{3-x}@C Nanocorn Electrocatalyst for Efficient CO_2 Reduction to HCOOH

Chen Qiu[1, #], Kun Qian[2, #] Jun Yu[1, *], Mingzi Sun[3], Shoufu Cao[4], Jinqiang Gao[1], Rongxing Yu[1, 5], Lingzhe Fang[2], Youwei Yao[5], Xiaoqing Lu[4], Tao Li[2, 6, *], Bolong Huang[3, *], Shihe Yang[1, 7, *]

[1]Guangdong Key Lab of Nano-Micro Material Research, School of Chemical Biology and Biotechnology, Peking University Shenzhen Graduate School, Shenzhen 518055, P. R. China

[2]Department of Chemistry and Biochemistry, Northern Illinois University, DeKalb, Illinois 60115, USA

[3]Department of Applied Biology and Chemical Technology, The Hong Kong Polytechnic University, Hung Hom, Kowloon, Hong Kong SAR, P. R. China

[4]School of Materials Science and Engineering, China University of Petroleum, Qingdao, Shandong 266580, P. R. China

[5]Shenzhen International Graduate School, Tsinghua University, Shenzhen, P. R. China

[6]X-ray Science Division and Joint Center for Energy Storage Research, Argonne National Laboratory, Lemont, Illinois 60439, USA

[7]Institute of Biomedical Engineering, Shenzhen Bay Laboratory, Shenzhen 518107, P. R. China

[#] Chen Qiu and Kun Qian contributed equally to this work.

*Corresponding authors. E-mail: yu.jun@pku.edu.cn (Jun Yu); taoli@aps.anl.gov (Tao Li); bhuang@polyu.edu.hk (Bolong Huang); chsyang@pku.edu.cn (Shihe Yang)

Supplementary Figures and Tables

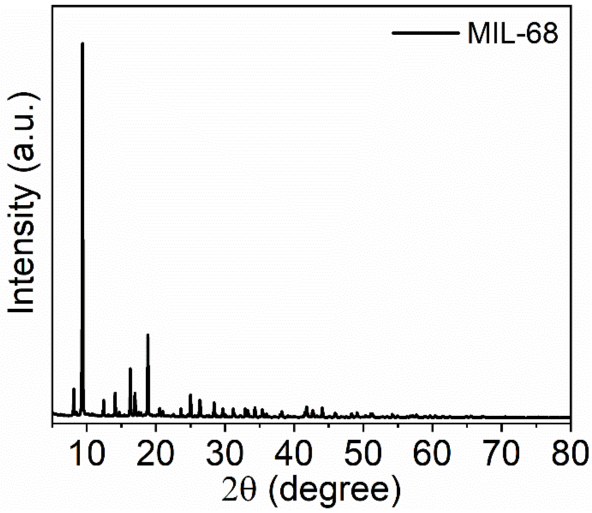

Fig. S1 X-ray diffraction (XRD) spectrum of the MIL-68 (In) sample

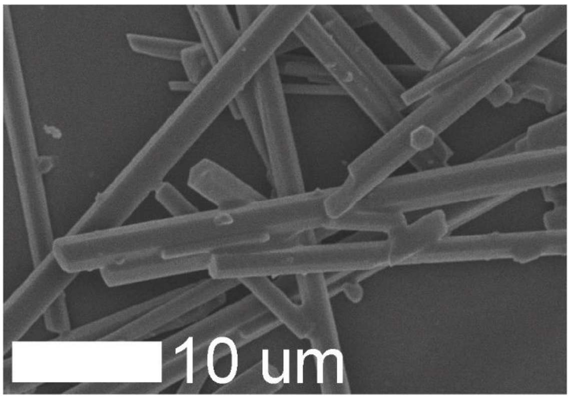

Fig. S2 The SEM image of MIL-68

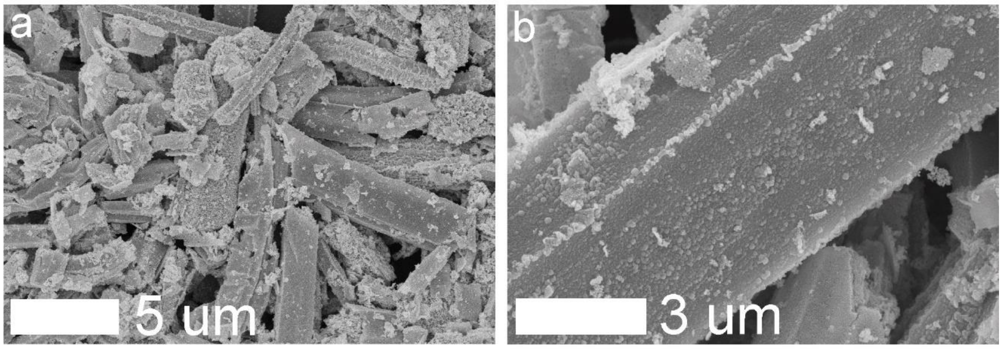

Fig. S3 The SEM images of MIL-68-Air

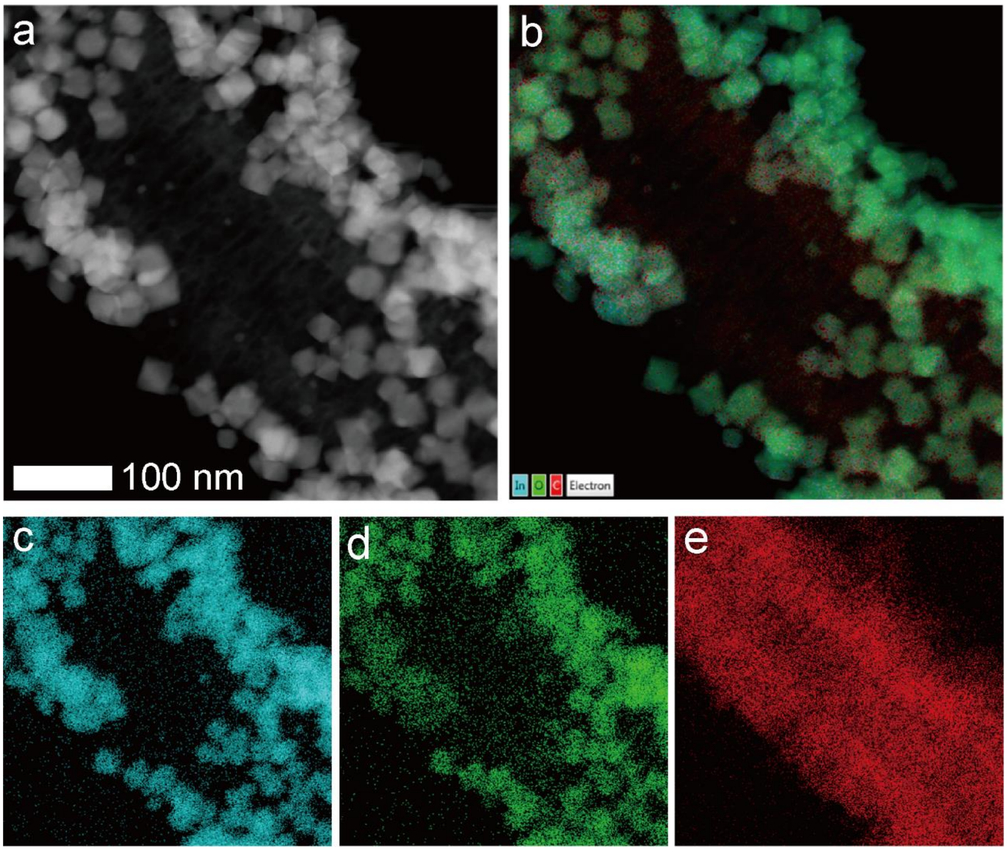

Fig. S4 a Transmission electron microscopy (TEM) image of the MIL-68-N_2 sample and the corresponding EDS mapping images of **b** all detected elements, **c** In element, **d** O element and **e** C element. Part of the surface cubic shape particles was removed to expose the beneath film through sonication

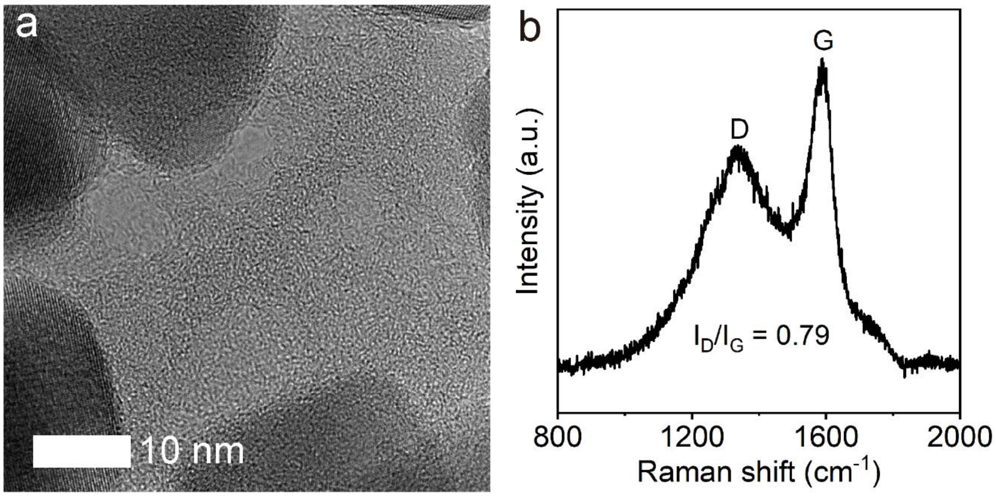

Fig. S5 a HR-TEM and **b** Raman spectrum of the MIL-68-N_2 sample

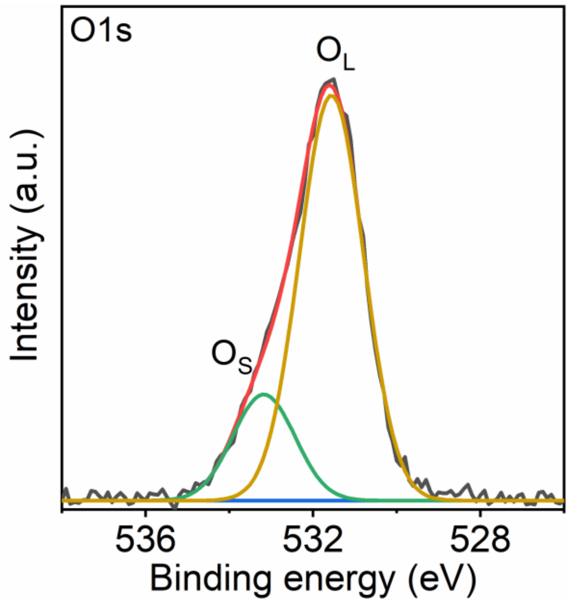

Fig. S6 O 1s XPS spectrum of the MIL-68-Air sample

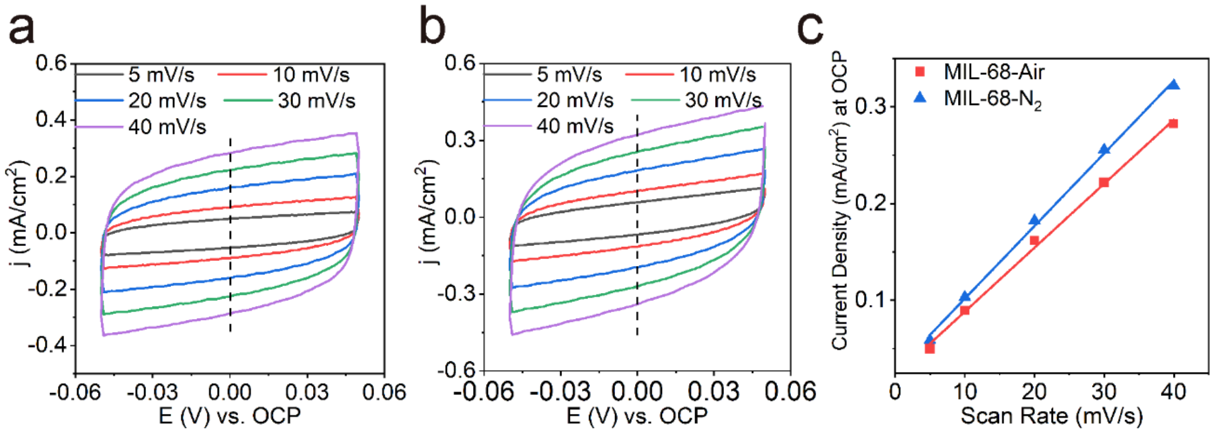

Fig. S7 Cyclic voltammograms of **a** MIL-68-Air and **b** MIL-68-N_2 measured in a non-Faradaic region of the voltammogram with the scan rates of 5, 10, 20, 30, and 40 mV s^{-1}. **c** Current density at OCP vs CV scan rate for the catalysts. The slope of current density at OCP vs scan rate represents the double-layer capacitance

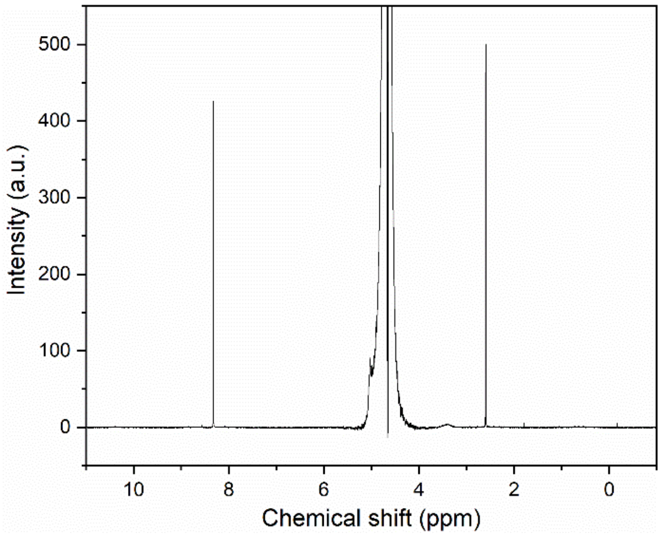

Fig. S8 Representative nuclear magnetic resonance (NMR) spectrum of the catholyte, peak at 8.3 ppm, 4.7 ppm and 2.6 ppm were ascribed to formate, water and DMSO, respectively

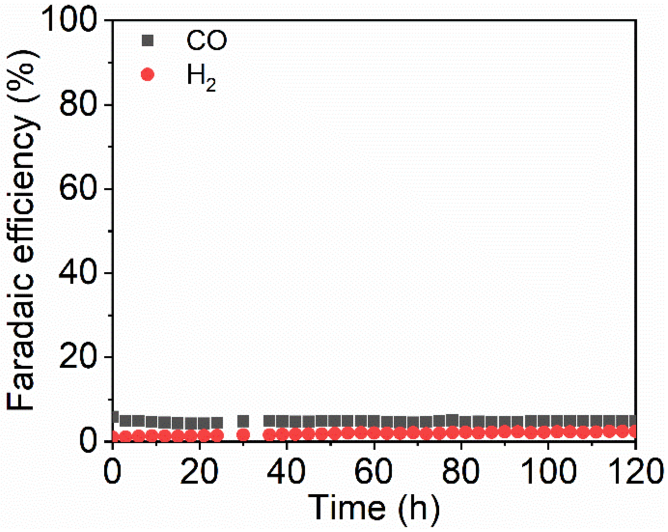

Fig. S9 FE of CO and H_2 during the stability test of the MIL-68-N_2 catalyst at the current density of 100 mA cm^{-2} for more than 120 h

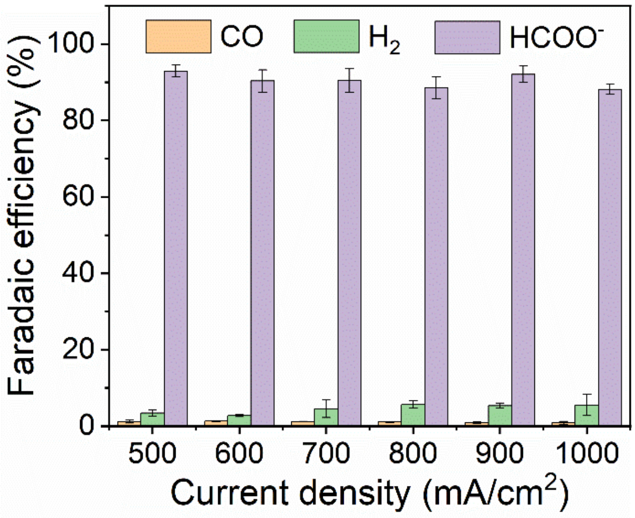

Fig. S10 Product distributions in terms of FE for the fixed current density of 500, 600, 700, 800, 900 and 1000 mA cm^{-2}

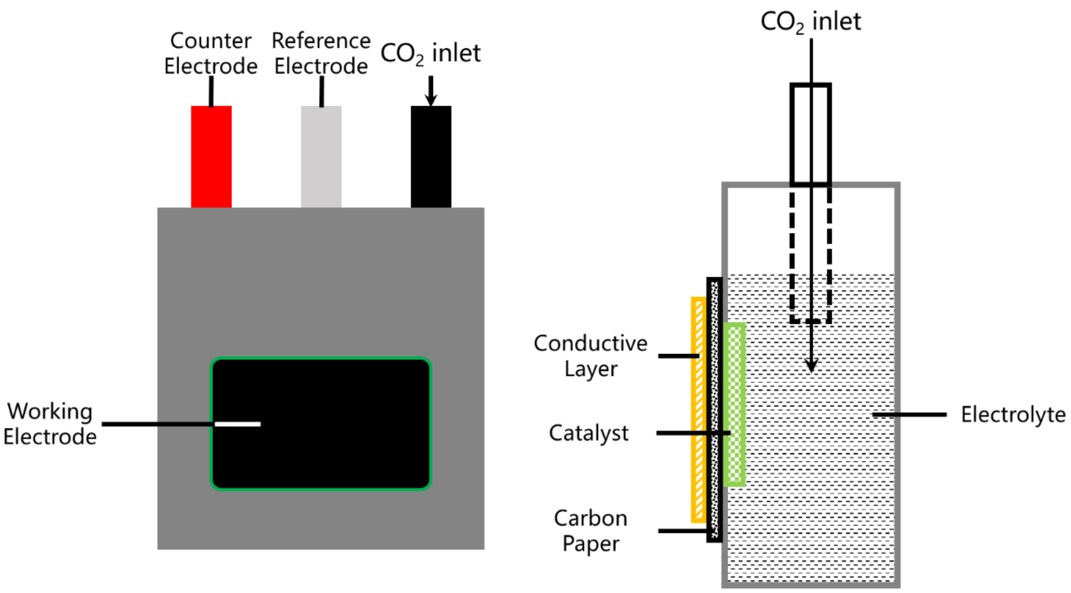

Fig. S11 Diagram of the *in situ* electrochemical cell

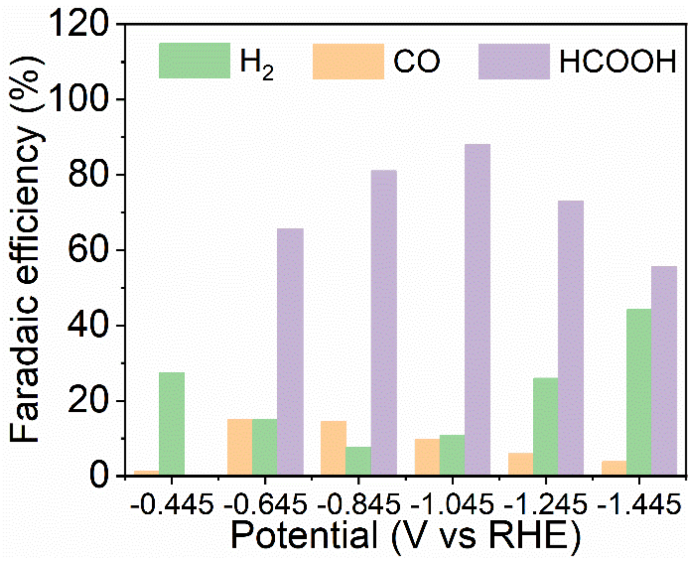

Fig. S12 FE and the product distribution vs the applied potentials for the MIL-68-N_2 catalyst tested in H-Cell. The electrolyte was 0.5 M $KHCO_3$ aqueous solution

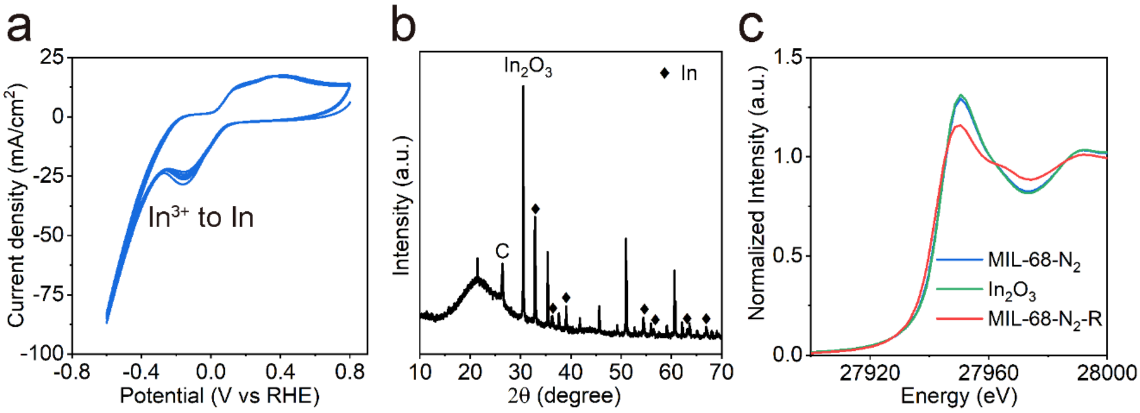

Fig. S13 a CV curves of the MIL-68-N_2 catalyst. **b** XRD pattern and **c** In K-edge XANES spectrum of the MIL-68-N_2 catalyst after electrolysis

Table S1 Summary of the current density and faradaic efficiency of HCOOH of our results and recently published data

**Catalyst**	**Reactor**	**Electrolyte**	**Applied potential /V_{RHE}**	**J_{HCOOH} /mA cm^{-2}**	**FE/%**	**Refs.**
In_2O_{3-x}@C	Flow cell	1 M KOH	-0.4 V	11*	84	This work
In_2O_{3-x}@C	Flow cell	1 M KOH	-1.0 V	218*	99	This work
In_2O_{3-x}@C	Flow cell	1 M KOH	-1.2 V	324*	99	This work
MIL-68(In)-NH_2	Flow cell	1 M KOH	-1.1 V	108*	94	[S1]
InP CQDs	Flow cell	1 M KOH	-2.5 V	368*	92	[S2]
hp-In	Flow cell	0.1 M $KHCO_3$	-1.1 V	45	90	[S3]
In/In_2O_{3-x}	H-cell	0.5 M $NaHCO_3$	-0.82 V	Low	89	[S4]
MFM-300(In)-e/In	H-cell	0.5 M $EmimBF_4$/MeCN	-2.15 $V_{Ag/Ag+}$	46	99	[S5]
$Cu_{25}In_{75}$	H-cell	0.5 M $NaHCO_3$	-0.7 V	Low	84	[S6]
In_2O_3-rGO	H-cell	0.1 M $KHCO_3$	-1.2 V	22	85	[S7]
H-InO_x	H-cell	0.5 M $NaHCO_3$	-0.7 V	Low	92	[S8]
MoP@In-PC	H-cell	[Bmim]PF_6 (30 wt%)/MeCN/H_2O(5wt%)	-2.2 $V_{Ag/Ag+}$	42	97	[S9]
$CuBi_2O_3$-PE	HFGDE	0.5 M $KHCO_3$	-1.0 V	120	85	[S10]
Bi nanosheets	Flow cell	0.5 M $KHCO_3$	-0.7 V<br>-0.9 V	Low<br>16	100<br>50	[S11]
Bi_2O_3@C	Flow cell	1 M KOH	-1.0 V	170*	93	[S12]
SnS	Flow cell	1 M KOH	-1.3 V	120*	88	[S13]

*stands for the current density without iR correction.
Low stands for the current density below 10 mA cm^{-2}

Table S2 The fitting parameters of In-O in In K-edge XANES spectra

**Sample**	**R (Å)**	**CN**	**σ2 (Å2)**	**ΔE (eV)**	**R factor**
**fresh**	2.17 ± 0.01	5.03 ± 0.29	0.005	3.8	0.00763
**CVs**	2.16± 0.01	3.97±0.28	0.00545	4.61	0.0153
**-0.445**	2.16±0.01	3.6±0.42	0.00511	3.46	0.0116
**-0.845**	2.17±0.01	4.93±0.49	0.0042	3.85	0.0271
**-1.045**	2.17±0.01	5.00±0.55	0.00419	4.1	0.01216
**-1.245**	2.175±0.01	4.64±0.54	0.00366	3.39	0.03983
**-1.445**	2.167±0.01	4.88±0.25	0.00396	2.52	0.00581

Supplementary References

[S1] Z. Wang, Y. Zhou, C. Xia, W. Guo, B. You et al., Efficient electroconversion of carbon dioxide to formate by a reconstructed amino-functionalized indium-organic framework electrocatalyst. Angew. Chem. Int. Ed. **60**(35), 19107-19112 (2021). https://doi.org/10.1002/anie.202107523

[S2] I. Grigioni, L.K. Sagar, Y.C. Li, G. Lee, Y. Yan et al., CO_2 electroreduction to formate at a partial current density of 930 mA cm^{-2} with inp colloidal quantum dot derived catalysts. ACS Energy Lett. **6**(1), 79-84 (2020). https://doi.org/10.1021/acsenergylett.0c02165

[S3] W. Luo, W. Xie, M. Li, J. Zhang, A. Züttel, 3D hierarchical porous indium catalyst for highly efficient electroreduction of CO_2. J. Mater. Chem. A **7**(9), 4505-4515 (2019). https://doi.org/10.1039/c8ta11645h

[S4] Y. Liang, W. Zhou, Y. Shi, C. Liu, B. Zhang, Unveiling in situ evolved In/In_2O_3- heterostructure as the active phase of In_2O_3 toward efficient electroreduction of CO_2 to formate. Sci. Bull. **65**(18), 1547-1554 (2020). https://doi.org/10.1016/j.scib.2020.04.022

[S5] X. Kang, B. Wang, K. Hu, K. Lyu, X. Han et al., Quantitative electro-reduction of CO_2 to liquid fuel over electro-synthesized metal-organic frameworks. J. Am. Chem. Soc. **142**(41), 17384-17392 (2020). https://doi.org/10.1021/jacs.0c05913

[S6] M. Zhu, P. Tian, J. Li, J. Chen, J. Xu et al., Structure-tunable copper-indium catalysts for highly selective CO_2 electroreduction to co or hcooh. ChemSusChem **12**(17), 3955-3959 (2019). https://doi.org/10.1002/cssc.201901884

[S7] Z. Zhang, F. Ahmad, W. Zhao, W. Yan, W. Zhang et al., Enhanced electrocatalytic reduction of CO_2 via chemical coupling between indium oxide and reduced graphene oxide. Nano Lett. **19**(6), 4029-4034 (2019). https://doi.org/10.1021/acs.nanolett.9b01393

[S8] J. Zhang, R. Yin, Q. Shao, T. Zhu, X. Huang, Oxygen vacancies in amorphous InO_x nanoribbons enhance CO_2 adsorption and activation for CO_2 electroreduction. Angew. Chem. Int. Ed. **58**(17), 5609-5613 (2019). https://doi.org/10.1002/anie.201900167

[S9] X. Sun, L. Lu, Q. Zhu, C. Wu, D. Yang et al., Mop nanoparticles supported on indium-doped porous carbon: outstanding catalysts for highly efficient CO_2 electroreduction. Angew. Chem. Int. Ed. **57**(9), 2427-2431 (2018). https://doi.org/10.1002/anie.201712221

[S10] H. Rabiee, L. Ge, X. Zhang, S. Hu, M. Li et al., Shape-tuned electrodeposition of bismuth-based nanosheets on flow-through hollow fiber gas diffusion electrode for high-efficiency CO_2 reduction to formate. Appl. Catal. B **286**, 119945 (2021). https://doi.org/10.1016/j.apcatb.2021.119945

[S11] L. Yi, J. Chen, P. Shao, J. Huang, X. Peng et al., Molten-salt-assisted synthesis of bismuth nanosheets for long-term continuous electrocatalytic conversion of CO_2 to formate. Angew. Chem. Int. Ed. **59**(45), 20112-20119 (2020). https://doi.org/10.1002/anie.202008316

[S12] P. Deng, F. Yang, Z. Wang, S. Chen, Y. Zhou et al., Metal-organic framework-derived carbon nanorods encapsulating bismuth oxides for rapid and selective CO_2 electroreduction to formate. Angew. Chem. Int. Ed. **59**(27), 10807-10813 (2020). https://doi.org/10.1002/anie.202000657

[S13] J. Zou, C.Y. Lee, G.G. Wallace, Boosting formate production from CO_2 at high current densities over a wide electrochemical potential window on a SnS catalyst. Adv. Sci. **8**(15), e2004521 (2021). https://doi.org/10.1002/advs.202004521
